# Supplementary material for: Altered microRNA Transcriptome in Cultured Human Liver Cells upon Infection with Ebola Virus
Source: Int J Mol Sci. 2021 Apr 6;22(7):3792. doi: 10.3390/ijms22073792 (PMC8038836; doi:10.3390/ijms22073792)
Supplement: Supplementary file 1 [file ijms-22-03792-s001.zip › Supplementary_File/C_ GO_Analysis_Results/16-30nt_go_Reston-96h-Huh7_vs_Control-96h-Huh7_down.mature_mirna_targets/MF_EnrichmentScore.pdf]

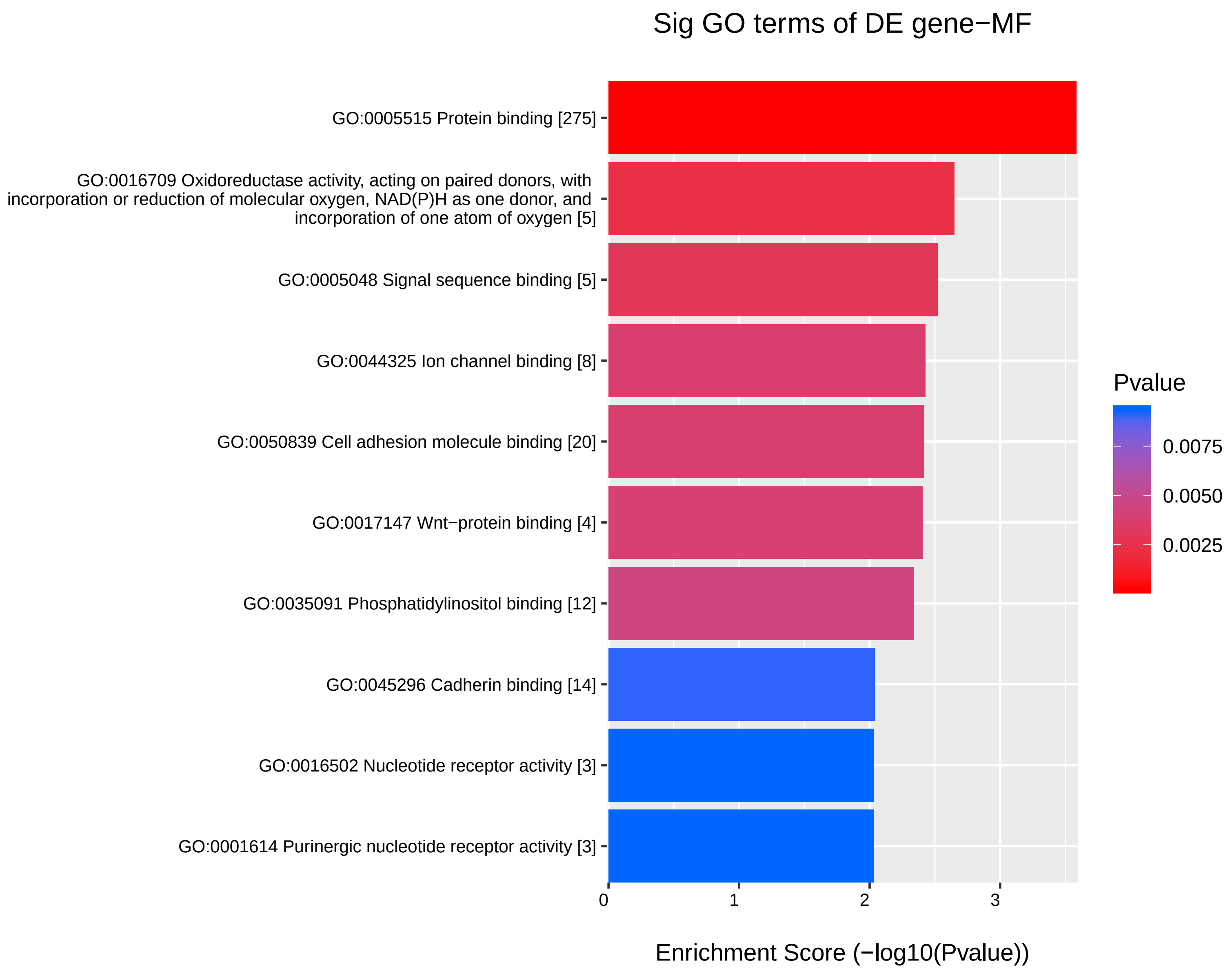
Sig GO terms of DE gene−MF
GO:0005515 Protein binding [275]
GO:0016709 Oxidoreductase activity, acting on paired donors, with incorporation or reduction of molecular oxygen, NAD(P)H as one donor, and incorporation of one atom of oxygen [5]
GO:0005048 Signal sequence binding [5]
GO:0044325 Ion channel binding [8]
GO:0050839 Cell adhesion molecule binding [20]
GO:0017147 Wnt−protein binding [4]
GO:0035091 Phosphatidylinositol binding [12]
GO:0045296 Cadherin binding [14]
GO:0016502 Nucleotide receptor activity [3]
GO:0001614 Purinergic nucleotide receptor activity [3]
0
1
2
3
Enrichment Score (−log10(Pvalue))
Pvalue
0.0075
0.0050
0.0025
